# Child sexual abuse presenting to a teaching hospital in colombo, Sri Lanka

**DOI:** 10.1192/j.eurpsy.2021.1679

**Published:** 2021-08-13

**Authors:** Y. Rohanachandra, I. Amarabandu, P.B. Dassanayake

**Affiliations:** 1 Department Of Psychiatry, University of Sri Jayewardenepura, Nugegoda, Sri Lanka; 2 Office Of The Judicial Medical Officer, Colombo South Teaching Hospital, Nugegoda, Sri Lanka

**Keywords:** sexual abuse, patterns of disclosure, victim characteristics

## Abstract

**Introduction:**

Child sexual abuse is a major public health problem in Sri Lanka, with prevalence rates ranging from 14-44%.

**Objectives:**

We aimed to describe the victim and perpetrator characteristics, pattern of disclosure and psychological consequences of sexual abuse in children presenting to a tertiary care hospital in Sri Lanka.

**Methods:**

This was a retrospective file review study of 164 victims who presented to a Teaching Hospital in Colombo, Sri Lanka, with alleged sexual abuse over a period of 5 years from 2015-2019.

**Results:**

Majority of the victims were female and older than 12 years. Majority (73.6%) have been subjected to penetrative sexual abuse with 58.5% of victims reporting more than one incident of abuse. Almost all (99.9%) of the perpetrators were male, with 94.5% being known to the child. Only 42.7% (n=70) of the children revealed about the incident within the first week. Delayed disclosure (i.e. more than 1 week since the incident) was significantly higher in penetrative abuse (p<0.01), multiple incidents of abuse (p<0.01) and in abuse by a known person (p<0.05). Children who disclosed after one week were significantly less likely to disclose about the incident spontaneously (p<0.01). Psychological sequel was seen in 28.7%, with depression being the commonest diagnosis (8.5%). Psychological consequences were significantly in higher those who had physical evidence of abuse (p<0.01), delayed (after 1 week) disclosure (p<0.05) and in those who did not disclose spontaneously (p<0.01).

**Conclusions:**

The victim and perpetrator characteristics, pattern of disclosure is comparable with previous literature.

**Disclosure:**

No significant relationships.

